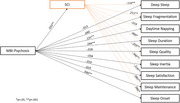# Sleep‐Related Disturbances, Psychosis, and Cognitive Decline in Healthy Older Adults: A Cross Sectional Analysis of PROTECT Study Data

**DOI:** 10.1002/alz70857_106113

**Published:** 2025-12-25

**Authors:** Satyam Chauhan, Ikran Dahir, Veena Kumari, Dag Aarsland, Clive G Ballard, Anne Corbett, Byron Creese

**Affiliations:** ^1^ Brunel University of London, London, Uxbridge, United Kingdom; ^2^ Brunel University of London, London, United Kingdom; ^3^ Centre for Healthy Brain Ageing, IoPPN, King's College London, London, United Kingdom; ^4^ University of Exeter, Exeter, United Kingdom

## Abstract

**Background:**

Sleep‐related disturbances are commonly reported in approximately 50% of older adults and have been associated with various psychiatric (e.g., psychosis) and neurodegenerative disorders (e.g., dementia). While the overlapping relationships between psychosis and sleep‐related disturbances in cognitive impairment have been recognised for decades, the mediating role of subjective cognitive impairment (SCI) in the relationship between MBI‐psychosis and sleep‐related disturbance is poorly understood. This study, therefore, aimed to investigate the magnitude of MBI‐psychosis on various sleep facets (i.e., sleep fragmentation, duration, inertia, quality, maintenance, satisfaction, daytime napping, deep sleep) while accounting for SCI in healthy UK‐based 14,846 older adults (74.3% females; mean age: 63.15±7.44).

**Method:**

All participants were recruited from the general population and completed a range of online self‐report measures on sleep, MBI‐psychosis, SCI. Linear regression was used to analyse the relationship between MBI‐psychosis and sleep; mediation analysis examined the effects of SCI on this relationship.

**Result:**

The findings demonstrated significant yet small‐sized correlations between MBI‐psychosis, SCI, and all sleep facets (*p* < 0.001). While MBI‐psychosis had a direct significant effect on all sleep facets; SCI also partially mediated the psychosis‐sleep relationship (*p* < 0.001). However, after controlling for depression, anxiety, and sex, the direct effect of psychosis on sleep was limited to sleep duration, daytime napping, and sleep onset. At the same time, SCI was found to fully mediate the relationship of psychosis with deep sleep, sleep fragmentation, inertia, quality, satisfaction and maintenance; SCI also partially mediated the relationship of psychosis with sleep onset and duration (all *β<0.2)*.

**Conclusion:**

These findings highlight the importance of early identification of sleep‐related disturbances and associated comorbid disorders, including depression and anxiety, in middle‐aged and older adults as a preventative strategy for cognitive decline and the onset of dementia.